# Supplementary material for: Causal Relationship between Aging and Anorexia Nervosa: A White-Matter-Microstructure-Mediated Mendelian Randomization Analysis
Source: Biomedicines. 2024 Aug 16;12(8):1874. doi: 10.3390/biomedicines12081874 (PMC11351342; doi:10.3390/biomedicines12081874)
Supplement: Supplementary file 1 [file biomedicines-12-01874-s001.zip › Supplementary Figure/Figure S4/Singe SNP plot of MR analysis for TL to ubm-b-1480.pdf]

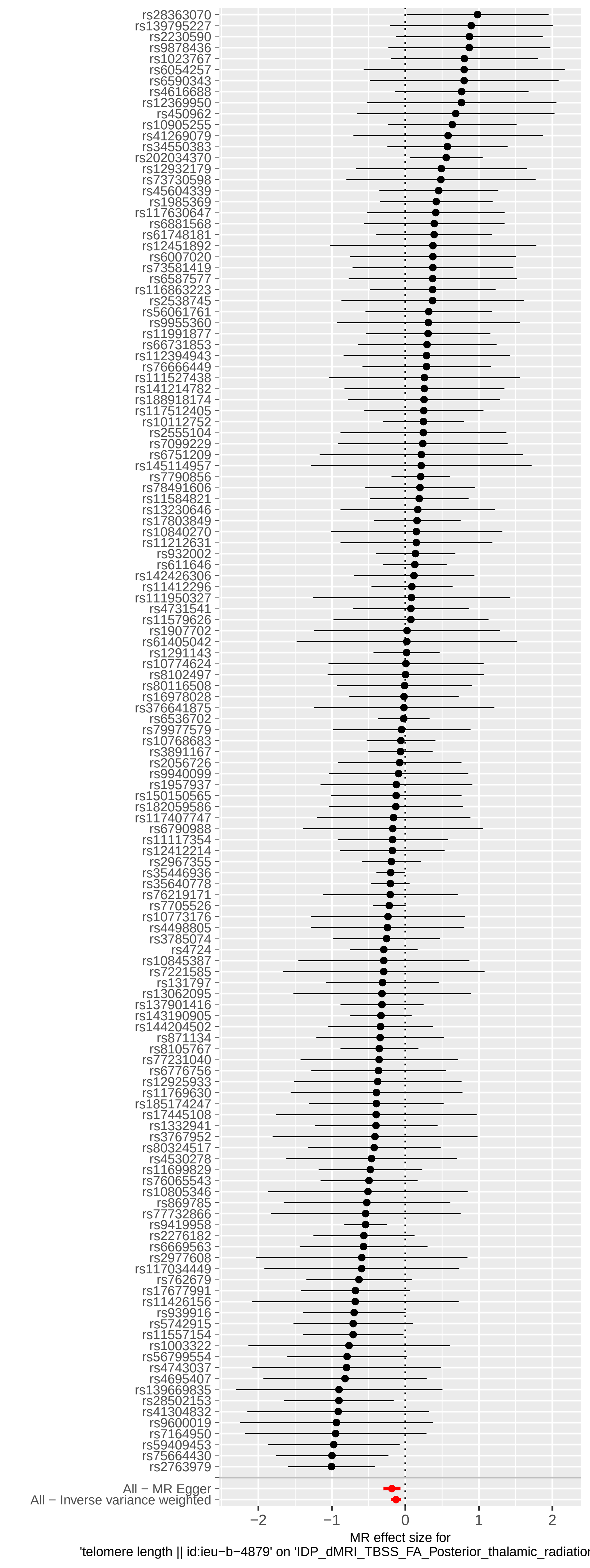
rs28363070
rs139795227
rs2230590
rs9878436
rs1023767
rs6054257
rs6590343
rs4616688
rs12369950
rs450962
rs10905255
rs41269079
rs34550383
rs202034370
rs12932179
rs73730598
rs45604339
rs1985369
rs117630647
rs6881568
rs61748181
rs12451892
rs6007020
rs73581419
rs6587577
rs116863223
rs2538745
rs56061761
rs9955360
rs11991877
rs66731853
rs112394943
rs76666449
rs111527438
rs141214782
rs188918174
rs117512405
rs10112752
rs2555104
rs7099229
rs6751209
rs145114957
rs7790856
rs78491606
rs11584821
rs13230646
rs17803849
rs10840270
rs11212631
rs932002
rs611646
rs142426306
rs11412296
rs111950327
rs4731541
rs11579626
rs1907702
rs61405042
rs1291143
rs10774624
rs8102497
rs80116508
rs16978028
rs376641875
rs6536702
rs79977579
rs10768683
rs3891167
rs2056726
rs9940099
rs1957937
rs150150565
rs182059586
rs117407747
rs6790988
rs11117354
rs12412214
rs2967355
rs35446936
rs35640778
rs76219171
rs7705526
rs10773176
rs4498805
rs3785074
rs4724
rs10845387
rs7221585
rs131797
rs13062095
rs137901416
rs143190905
rs144204502
rs871134
rs8105767
rs77231040
rs6776756
rs12925933
rs11769630
rs185174247
rs17445108
rs1332941
rs3767952
rs80324517
rs4530278
rs11699829
rs76065543
rs10805346
rs869785
rs77732866
rs9419958
rs2276182
rs6669563
rs2977608
rs117034449
rs762679
rs17677991
rs11426156
rs939916
rs5742915
rs11557154
rs1003322
rs56799554
rs4743037
rs4695407
rs139669835
rs28502153
rs41304832
rs9600019
rs7164950
rs59409453
rs75664430
rs2763979
All − MR Egger
All − Inverse variance weighted
−2
−1
0
1
2
MR effect size for
'telomere length || id:ieu−b−4879' on 'IDP_dMRI_TBSS_FA_Posterior_thalamic_radiatio
